# Baiting improves CPUE in nine-spined stickleback (*Pungitius pungitius*) minnow trap fishery

**DOI:** 10.1002/ece3.1635

**Published:** 2015-08-18

**Authors:** Juha Merilä

**Affiliations:** Department of Biosciences, Ecological Genetics Research Unit, University of HelsinkiP.O. Box 65, Helsinki, FI-00014, Finland

**Keywords:** Bait, CPUE, *Haemopis sanguisuga*, minnow trap, ninespine stickleback, *Pungitius pungitius*

## Abstract

Whether or not baiting influences stickleback catch per unit effort (CPUE) remains a matter of debate among stickleback researchers: While the opinions about the impact of baiting on CPUE differ, supporting quantitative data are scarce. The effect of baiting and trap type on nine-spined stickleback (*Pungitius pungitius*) CPUE was studied in a field experiment conducted over four consecutive days in a small pond in northeastern Finland. The results show that baited traps yielded better (mean CPUE = 1.24 fish/trap/d) catches than unbaited traps (mean CPUE = 0.66); however, there were also differences in CPUE depending on the type of collapsible trap that was used. The trap type effect on CPUE seemed to differ among age classes – the finer meshed trap caught more young-of-the-year fish than the coarse-meshed one, whereas the opposite was true for the older and larger individuals. The results agree with those of an earlier more restricted study conducted in the same locality: Together, these results provide strong evidence for the positive impact of baiting on nine-spined stickleback CPUE.

## Introduction

Apart from the three-spined stickleback (*Gasterosteus aculeatus*) fishery for the purposes of fish oil production and/or poultry forage (e.g., Järvi 1932; Ojaveer 1999), the interest toward sticklebacks as a fishery target has received little attention in the scientific literature. This is in spite of the fact that a large worldwide community of researchers has utilized sticklebacks as models in their scientifically diverse research since the 1960s (for reviews, see: Bell and Foster 1994; Östlund-Nilsson et al. 2007; von Hippel 2010; Wootton 1976, 1984, 2009; Merilä 2013). Given the logistic challenges of maintaining sticklebacks in the laboratory over longer periods of time, this scientific community often obtains their study materials mainly from yearly seine, dipnet, or minnow trap catches. Among the scientists involved in the stickleback fishery, opinions about the effectiveness of various gear and baiting differ. Yet, very little systematic effort has been directed toward comparing the efficiency of different gear and in particular whether baiting has an influence on CPUE (but see: Merilä 2012; Merilä et al. 2012; Merilä 2015).

Baiting of traps and fishing gear is known to be an effective way of improving CPUE of many different fish species (von Brandt 1984; Stoner 2004). However, the effectiveness of baiting may be highly context dependent and differ depending on the prevailing abiotic (e.g., temperature) and/or biotic (e.g., predators, competitors) environmental conditions that are known to influence fish behavior (cf. activity, feeding capability, and motivation) and thereby also catchability (Stoner 2004). Consequently, the effect of baiting on CPUE may differ even among ecologically similar species (Løkkeborg et al. 1989; Furevik and Løkkeborg 1994) and also within a given target population depending on the prevailing conditions (e.g., Bigelow et al. 1999; Stoner 2004; Dupuch et al. 2011).

While experimental work on the three-spined stickleback has found no evidence that baiting of traps would improve CPUE (Merilä 2015), evidence from the nine-spined stickleback (*Pungitius pungitius*) suggests the opposite (Merilä 2012). Namely, in a comparison of baited and unbaited minnow traps, Merilä (2012) found that baiting significantly improved nine-spined stickleback CPUE. However, the result was based on catches from a single trapping occasion, and given the multitude of extrinsic factors that influence fish behavior and CPUE, confirmatory evidence for the positive effect of baiting on CPUE is lacking.

The main aim of this study was to seek confirmatory evidence for the earlier suggestion (cf. Merilä 2012) that baiting of minnow traps improves their CPUE in the nine-spined stickleback fishery. In addition, the impact of two different trap types on CPUE was investigated, along with the influence of baiting and trap type on CPUE of different sized (*viz*. giant vs. young of the year) individuals.

## Material and Methods

The experiments were carried out on four consecutive days between the 7th and 11th of July 2012, in the small (<0.05 km^2^ surface area, max. depth of *ca*. 5.2 m) Rytilampi lake in northeastern Finland (66°23′N, 29°19′E), where earlier studies (Merilä 2012; Merilä et al. 2012) focusing on nine-spined stickleback minnow trap catches have been conducted. Rytilampi is an oligotrophic lake in which the nine-spined stickleback is the only fish species present. Individuals in this locality can reach relatively old ages (up to 7 years; DeFaveri et al. 2014) and “giant” sizes (>115 mm in total length; Herczeg et al. 2009).

Nine “sets” of collapsible minnow traps were deployed. Seven of the sets consisted of two different kinds of traps: three “coarse-meshed black” (Promar [Gardena, CA] TR-503; mesh size 9 mm) and three “fine-meshed brown” (Promar TR-501; mesh size 2 mm) traps made of polyethylene netting. These are the same traps that were used in Merilä (2012), from where details and photographs of their dimensions can be found. Due to a lack of sufficient numbers of fine-meshed brown traps, the two remaining sets consisted only of the coarse-meshed black traps. Only three traps were used in each of these sets (as compared to the total of six traps used in the seven sets described above). In the seven sets with both trap types, two traps – one of each type – were left as controls, whereas two (one of each type) were baited with about 10 g of blue cheese grains (Valio, Finland), and two (one of each type) were fitted with an aluminum foil “attractor”, exactly as described in Merilä (2012). The rationale behind the aluminum foil was to test whether reflections would attract fish to the traps – an idea born from the observation that “silvery” galvanized minnow traps catch more nine-spined sticklebacks than matte black nylon-coated ones of similar size and shape (Merilä et al. 2012). In the two sets that contained only the coarse-meshed black traps, each of the three traps within a set was allocated to one of the three treatments as described above. Hence, a total of 48 traps (27 coarse-meshed black and 21 fine-meshed brown) were fished every day. The traps within each set were deployed about 1–2 m apart from each other, and the distance between the different sets varied from *ca*. 30 to 300 m.

The traps were set from shore (about 1–2 m off the shoreline) in late afternoon of July 7th and checked every 24 h over the next four consecutive days. The number of fish in the traps was counted each day, and the size of the fish was judged by eye to be either (1) young of the year (total length < 25 mm) or (2) giants (total length > 80 mm). The intermediate-sized fish were a mixture of adults and immature individuals. All fish were released back to the site of their capture after they had been counted.

Horseleeches (*Haemopis sanguisuga*) – which can grow up to 150 mm in length – are believed to prey mostly on invertebrates (e.g., Sawyer 1986; Shikov 2011), but over the course of the fieldwork in Rytilampi lake, I have observed them feeding also on sticklebacks, especially in traps. Hence, in order to evaluate whether stickleback CPUE could be influenced by horseleeches, their numbers in the traps were also counted.

The data were analyzed with repeated measures analysis of variance, treating log10-transformed catches (number of fish + 1) at four different time points as time-dependent response variables. Separate models were conducted for the total catch, giants, and juveniles. Horseleech catches were also analyzed separately. As the experimental design was not entirely balanced (cf. both trap types not present in all sets; see above), only the main effects of set, trap type, and treatment as between-subject factors (but not their interactions) were fitted. Time (i.e., different trapping days) and its interactions with the between-subject factors were fitted as within-subject effects. Although residuals of most fitted models were normally or nearly normally distributed, some deviations occurred (Shapiro–Wilk W-tests), and hence, the assumptions of parametric tests were sometimes violated. Therefore, to assess the robustness of the main results in regard to baiting and trap type effects on CPUE, a series of confirmatory nonparametric tests were also conducted.

First, to test the effect of trap type on CPUE, the catches from each trap over the 4 days were first summed and the CPUE across the treatments was normalized by running a one-way ANOVA with treatment as a factor. The residuals from this model were used to test for trap type effect with Wilcoxon's test (i.e., one-way ANOVA based on rank scores). Second, the converse approach was also applied: The CPUE (summed over the 4 days) across the trap types was normalized using a one-way ANOVA, and the residuals from this model were subjected to a nonparametric multiple comparison test (Wilcoxon method) to test for the effect of treatment on CPUE. Concurring results from nonparametric and parametric tests are interpreted to verify that conclusions drawn from the latter are robust.

All the analyses were conducted with JMP (ver. 11.0.0; SAS Institute, Inc., Cary, NC) statistical package.

Because the work described in this study does not constitute an animal experiment in a legal sense, the only required permissions were national personal fishing license for 2012 and a license (# 3087/41/2011) from the owner (Metsähallitus) of the water body where the fishing was conducted.

## Results

A total of 161 sticklebacks were caught, with an average CPUE of 0.839 (SD = 1.228) fish/trap/d. Most (*n* = 116; 72%) of the caught fish were adults or immature individuals. About 16% (*n* = 45) of the fish were giants. Juveniles (young of the-year) were a clear minority (*n* = 26; 28% of the fish). A total of 128 horseleeches were caught (CPUE = 0.667, SE = 1.279 leeches/trap/d).

**Figure 1:**
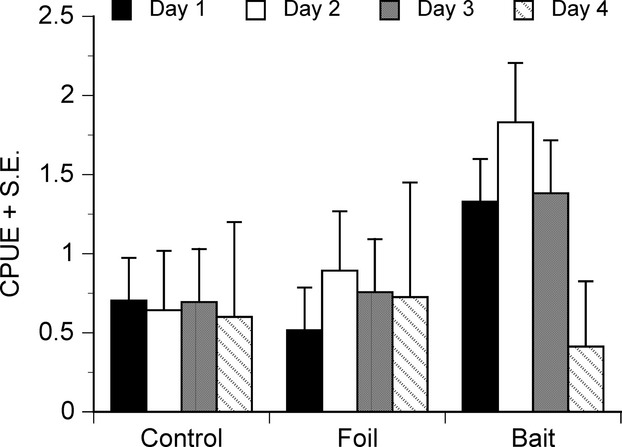
Effect of baiting on mean CPUE (+SE) of total catch of nine-spined sticklebacks on four different days. Values are least square means adjusted for effects of treatment and set on CPUE. For statistical tests, see Table1.

Repeated measures analyses revealed that total CPUE was significantly influenced by treatment (Table1a), with baited traps yielding a higher CPUE than the unbaited or foil traps (Fig.1). None of the other effects in the model were significant (Table1a). Results from the nonparametric tests were entirely concurrent with these results: Only the treatment effect was significant (Table2).

**Table 1:** Results of the multivariate repeated measures analyses of variance of log-transformed CPUEs

Response	Effect	*F*	Ndf, Ddf	*P*
(a) Total CPUE	Set	0.945	8,36	0.492
	Trap type	0.431	1,36	0.516
	Treatment	3.941	2,36	**0.028**
	Time	1.178	3,34	0.260
(b) Giant CPUE	Set	1.940	8,36	0.084
	Trap type	19.463	1,36	**<0.001**
	Treatment	2.626	2,36	0.086
	Time	0.364	3,34	0.779
(c) Juvenile CPUE	Set	1.972	8,36	0.079
	Trap type	23.401	1,36	**<0.001**
	Treatment	0.790	2,36	0.460
	Time	0.978	3,34	0.512
(d) Horseleech CPUE	Set	0.681	8,36	0.705
	Trap type	6.323	1,36	**0.016**
	Treatment	3.659	2,36	**0.036**
	Time	3.618	3,34	**0.023**

Ndf and Ddf, numerator and denominator degrees of freedom, respectively. *P-*values in bold are statistically significant.

**Table 2:** Results of nonparametric test for effects of trap type and treatment on CPUE of nine-spined sticklebacks (total, giant, and juvenile) and horseleeches. The tabled values are chi-square test values (df = 1) for effects of trap type and *z*-values for effects of treatment (see methods for further details)

Comparison	Total	Giant	Juveniles	Leeches
Trap type	1.83	21.27***	15.37***	6.96**
Treatment				
Control vs. Foil	−0.05	0.88	−0.40	−0.31
Control vs. Baited	2.12*	2.07*	0.66	2.81**
Foil vs. Baited	2.00*	1.09	1.00	2.08*

*P < 0.05, **P < 0.01, ***P < 0.001.

**Figure 2:**
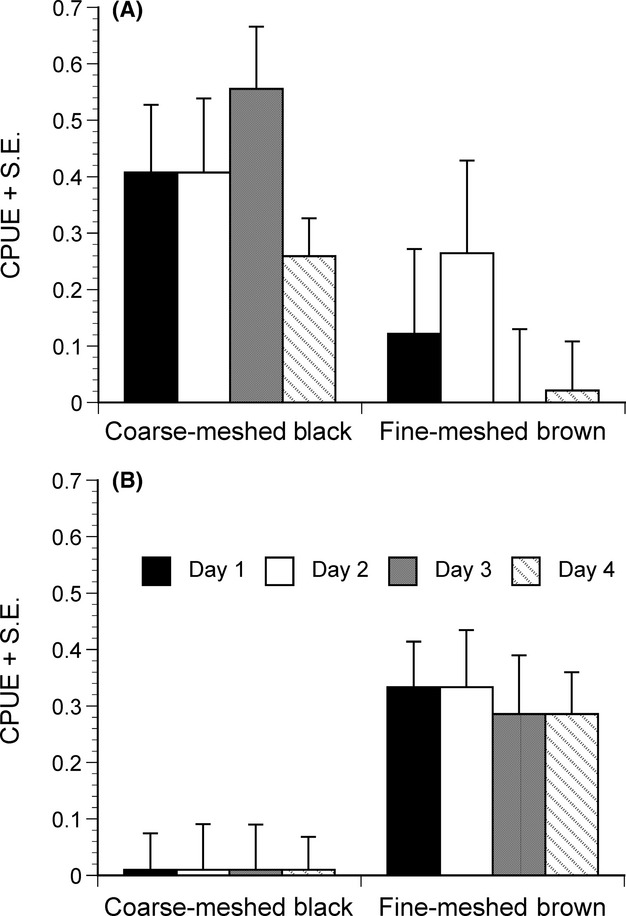
Effect of trap type on mean (+SE) CPUE of (A) giant and (B) juvenile nine-spined sticklebacks on four different days. Values are least square means adjusted for effects of treatment and set on CPUE. For statistical tests, see Table1.

Trap type had a significant effect on the CPUE of the giants (Table1b), with the coarse-meshed black traps catching more giants than the fine-meshed brown traps (Fig.2A). None of the other effects in the model were significant, albeit the effects of set and treatment (baited > foil ≈ control traps) were only marginally nonsignificant (Table1b). Again, the nonparametric test confirmed the significant effect of the trap type, and also the effect of baiting in one of the comparisons (baited > control) was significant (Table2).

Trap type also had a significant effect on CPUE of the juveniles (Table1c), with the fine-meshed brown traps catching more fish than the coarse-meshed black traps (Fig.2B). None of the other effects in the model were significant, albeit the effect of set was marginally nonsignificant (Table1c). Nonparametric tests confirmed the significant effect of trap type on juvenile CPUE and that the baiting did not seem to improve juvenile CPUE (Table2).

Horseleech CPUE was influenced both by trap type (fine-meshed brown > coarse-meshed black) and treatment, with more leeches caught from baited than from unbaited traps (Table1d). Again, the results of the nonparametric tests confirmed both of these results (Table2). The time effect in the repeated measures analysis was also significant (Table1d), and this came about due to the declining CPUE over the successive catches (*r*_s_ = −0.159, *P *=* *0.027). There was also a significant time*trap type interaction (*F*_3,34_ = 6.499, *P *=* *0.0014) in horseleech CPUE, and this was due to the fact that the CPUE for the two trap types was not consistent over the 4 days (results not shown).

## Discussion

The most salient finding of this study was the higher stickleback CPUE of baited minnow traps, as compared to those that were not baited. However, the baiting mainly seemed to influence the CPUE of older and larger nine-spined sticklebacks, but it did not have an effect on the CPUE of the young-of-the-year fish. The type of trap also had a clear impact on CPUE, where the black coarse-meshed traps caught more giants than the fine-meshed brown traps, while the opposite was true for the young-of-the-year fish. These results are largely in agreement with the earlier findings of Merilä (2012) and confirm – with a trapping effort over ten times higher – that baiting indeed improves nine-spined stickleback CPUE. The fact that baited traps also attracted more stickleback-preying horseleeches than the unbaited traps adds further strength to the conclusion that baiting improves stickleback CPUE. In other words, in spite of the leech predation, the effect of baiting was still detectable. In the following, these findings and their interpretations are discussed in relation to what is previously known about factors influencing CPUE in stickleback fisheries.

The results show that baiting had a positive effect on total nine-spined stickleback CPUE. This agrees with the initial findings of an earlier study from this species and locality (Merilä 2012), based on a much smaller sample size. Using data from Merilä (2012), I estimate that the positive effect of baiting on CPUE (measured as mean CPUE_baited traps_/CPUE_unbaited traps_) in the earlier study was 3.5-fold, while the effect on CPUE in this study was about 1.9-fold. The smaller effect in the current study can have various mutually nonexclusive explanations, including temporal differences in environmental conditions, population density, density of predators (cf. leeches), and also annual differences in age and size structure of the study population. For instance, as the results show here, the effect of baiting was not significant for juveniles. A higher abundance of juveniles in 2012 as compared to 2011 could have contributed to the lowered effect of baiting on CPUE estimates in 2012. Similarly, given that more horseleeches were caught from baited than from unbaited traps, they might have lowered stickleback CPUE more in 2012 than in 2011.

One should also note that the locality where both the earlier and current experiments were conducted is an oligotrophic lake, where food for sticklebacks is likely to be a limiting supply. Hence, while the positive impact of baiting on stickleback CPUE in this locality seems to be reproducible, it remains to be tested whether the results also hold in populations residing in different environmental conditions. Similar experiments with another stickleback species – the three-spined stickleback (*Gasterosteus aculeatus*) – conducted in the Baltic Sea did not find any effect of baiting on CPUE (Merilä 2015). However, whether this difference is species, population or habitat specific remains to be investigated.

While the trap type did not have any effect on the total stickleback CPUE, the coarse-meshed black traps caught more giants than the fine-meshed brown traps and *vice versa* in the case of the young-of-the-year fish. This difference in trap type-specific CPUE is understandable, as the brown traps are more fine-meshed and have smaller entrances than the coarse-meshed black traps. Hence, the fine-meshed brown traps are likely to be more effective in retaining small fish than the coarse-meshed black traps, and also, it is possible that the larger fish may more readily enter into the more “open” coarse-meshed black than to the more “closed” brown traps (Merilä 2012). Nevertheless, at least when it comes to catching adult and immature nine-spined sticklebacks, as well as adult three-spined sticklebacks, the coarse-meshed black traps seem to be more effective than the fine-meshed brown traps (Merilä 2012, 2015). That said, the metallic Gee traps might be even more effective in catching nine-spined sticklebacks than either of the collapsible minnow trap models used in this study. Merilä et al. (2012) noted that CPUEs from Gee traps from this locality exceeded that of collapsible minnow traps, but that CPUE comparison may only reflect spatial and environmental – rather than trap type-specific – differences, as the Gee traps were fished in deeper water than the collapsible Promar traps.

The average CPUE in this study (0.84 fish/trap/d) was about six times lower than that from the same locality the year before (CPUE = 4.86 fish/trap/d; Merilä 2012). How to reconcile this difference in light of the fact that the same methods were used at the same time of the year (2011: July 13–14th; 2012: July 7–11th)? Catch per unit effort depends both on catch efficiency and population abundance (e.g., Harley et al. 2001; Hubert and Fabrizio 2007), and difference in one or both of these factors might have differed among the two study years. Catch efficiency is mainly influenced by fish behavior, which in turn can be influenced by various environmental factors such as weather conditions and predators (Stoner 2004; Hubert and Fabrizio 2007; Lake 2013). While my data do not allow me to infer whether there was a difference in catch efficiency between two study years, I am inclined to think that the more likely explanation was lower stickleback abundance in 2012 as compared to 2011. Another possible explanation is the heavy horseleech predation in 2012 as compared to 2011, although quantitative data in this respect are lacking from 2011. More data would be needed to understand how CPUE in the stickleback minnow trap fishery is influenced by catch efficiency and population abundance. This could be done, for instance, in mesocosms where the actual abundance is known, but environmental conditions vary (or are manipulated) over time.

Finally, more horseleeches were caught from baited as compared to nonbaited traps. This effect could arise if the leeches are attracted to the fish (which were more abundant in the baited traps), or because the bait odor itself also attracts leeches independently of the fish in the traps. Both hypotheses are plausible, as leeches are known to have sensitive chemosensory and mechanosensory organs (Elliott 1986; Lent and Adams 1989; Simon and Barnes 1996), and thus, are able to orientate according to both chemical and tactical cues. Data from the present study do not allow differentiation between these two mutually exclusive hypotheses, but it is possible that leeches may have influenced the CPUE estimates by removing fish from the traps, or by repelling fish from entering into the traps. However, if the leeches had a strong negative influence on CPUE, one would have expected to see a negative correlation between fish and leech CPUEs. Given that both fish and leech CPUEs peaked in baited traps, it seems that if anything, leech predation on fish has reduced the CPUE difference between baited and unbaited traps. In other words, the effect of baiting on CPUE might have been underestimated due to leech predation.

The practical implication of this study for researchers aiming to catch nine-spined sticklebacks with minnow traps is that baiting is likely to improve CPUE. In addition, the choice of trap type seems to matter, where the coarse-meshed black traps may be the traps of choice if the target population is large-sized adult fish. However, given that galvanized Gee traps have been shown to yield high nine-spined stickleback CPUEs even without baiting (Merilä et al. 2012), they may provide an equally good if not better choice. However, given that the results of the current study were obtained from one particular locality at one particular time point, these recommendations may not yield improved catches in all situations. For instance, efficiency of baiting may vary spatially and seasonally and may also be sex dependent. Nevertheless, in the absence of better information, and when usage of a seine net is not an option (e.g., muddy lakes with many subemerged obstacles), baited minnow traps should provide, if not better, at least an equally good choice than unbaited ones.

In conclusion, the results confirm the earlier suggestion that baiting improves minnow trap CPUE in nine-spined stickleback fishery and that different trap models differ in their CPUE. Apart from studies seeking to test the effect of baiting on stickleback CPUE in other localities and environmental conditions, an interesting avenue for future research would be in evaluating whether or not horseleeches are actually the top predator (aside from piscivorous birds, such as divers [*Gavia* sp.]) in this particular study system, rather than opportunists predating only/mainly on fish caught in traps. One way to test this hypothesis would be to compare the levels of stable isotopes of *δ*^15^N in leeches and nine-spined sticklebacks: If the leeches regularly use sticklebacks as forage, they would be expected to show higher fractionation of *δ*^15^N as compared to nine-spined sticklebacks (e.g., Post 2002).
